# Excellent Ultracold Molecular Candidates From Group VA Hydrides: Whether Do Nearby Electronic States Interfere?

**DOI:** 10.3389/fchem.2021.778292

**Published:** 2021-12-16

**Authors:** Donghui Li, Wensheng Bian

**Affiliations:** ^1^ Beijing National Laboratory for Molecular Sciences, Institute of Chemistry, Chinese Academy of Sciences, Beijing, China; ^2^ School of Chemical Sciences, University of Chinese Academy of Sciences, Beijing, China

**Keywords:** molecular laser cooling, *ab initio*, spin-orbit coupling, group VA hydrides, electronic state crossing, ultracold molecules

## Abstract

By means of highly accurate *ab initio* calculations, we identify two excellent ultracold molecular candidates from group VA hydrides. We find that NH and PH are suitable for the production of ultracold molecules, and the feasibility and advantage of two laser cooling schemes are demonstrated, which involve different spin-orbit states ( $A^{3}\Pi_{2}$ and $X^{3}\Sigma_{1}^{-}$ ). The internally contracted multireference configuration interaction method is applied in calculations of the six low-lying Λ-S states of NH and PH with the spin-orbit coupling effects included, and excellent agreement is achieved between the computed and experimental spectroscopic data. We find that the locations of crossing point between the $A^{3}\Pi$ and Σ−5 states of NH and PH are higher than the corresponding *v*′ = 2 vibrational levels of the $A^{3}\Pi$ state indicating that the crossings with higher electronic states would not affect laser cooling. Meanwhile, the extremely small vibrational branching loss ratios of the $A^{3}\Pi_{2}$ → $a^{1}\Delta_{2}$ transition for NH and PH (NH: 1.81 × 10^–8^; PH: 1.08 × 10^–6^) indicate that the $a^{1}\Delta_{2}$ intermediate electronic state will not interfere with the laser cooling. Consequently, we construct feasible laser-cooling schemes for NH and PH using three lasers based on the $A^{3}\Pi_{2}$ → $X^{3}\Sigma_{1}^{-}$ transition, which feature highly diagonal vibrational branching ratio $R_{00}$ (NH: 0.9952; PH: 0.9977), the large number of scattered photons (NH: 1.04×10^5^; PH: 8.32×10^6^) and very short radiative lifetimes (NH: 474 ns; PH: 526 ns). Our work suggests that feasible laser-cooling schemes could be established for a molecular system with extra electronic states close to those chosen for laser-cooling.

## Introduction

Searching for promising laser cooling candidates to produce ultracold polar molecules has attracted considerable research interests in recent years owing to their importance for a lot of promising applications in various fields such as precision measurements, quantum computing and quantum information (Hudson et al., 2011; Yan et al., 2013; Baron et al., 2014). One of the most remarkable successes is direct laser cooling of SrF to the *µ*K level in 2010 (Shuman et al., 2010), which has initiated many research interests in molecular laser cooling. However, up to now only a very limited number of molecules have been successfully cooled to the ultracold temperatures experimentally. So there is an urgent necessity to search for more promising laser cooling candidates, and some theoretical efforts have been made to identify more candidates for laser cooling (Wells and Lane, 2011; Fu et al., 2017; Cao et al., 2019; Moussa et al., 2021). It is known (Fu et al., 2016; Yuan et al., 2019; Li et al., 2021) that, a suitable candidate for laser cooling needs to satisfy three criteria: highly diagonal Franck-Condon factors (FCFs), an extremely short radiative lifetime, and no interference from the intermediate electronic states. In our recent work, the fourth criterion for molecular laser cooling was proposed, that is, no electronic-state crossing, or the crossing point between the two states was high enough in energy (Li et al., 2020). Consequently, all electronic states close to those chosen for laser-cooling should be calculated and checked beforehand in selecting laser-cooling candidates.

Many studies have been performed for NH and PH over the past decades. Experimentally, most previous studies were based upon spectroscopic techniques. In 1959, Dixon (1959) observed the emission spectra of the $A^{3}\Pi$ → $X^{3}\Sigma^{-}$ transition of NH and photographed the (0, 0) and (1, 0) bands. In 1976, Smith et al. (1976) observed weak predissociation from the $A^{3}\Pi$ state of NH via high resolution lifetime measurements using the high-frequency deflection technique. In 1986, the emission spectra of the $A^{3}\Pi$ → $X^{3}\Sigma^{-}$ transition of NH were observed by Brazier et al. (1986) using a high-resolution Fourier transform spectrometer. They reported the vibrational, fine structure and rotational constants of the two states. In 1999, the high-resolution emission spectra of the $A^{3}\Pi$ → $X^{3}\Sigma^{-}$ transition of NH were observed using a Fourier transform spectrometer, and five vibration-rotation bands were measured (Ram et al., 1999). On the other hand, in 1974, the emission spectra of the $A^{3}\Pi$ → $X^{3}\Sigma^{-}$ transition of PH were photographed with high resolution, and the (0, 0) and (0, 1) bands were obtained (Rostas et al., 1974). In 1985, Gustafsson et al. (1985) recorded the emission spectra of the $A^{3}\Pi$ → $X^{3}\Sigma^{-}$ transition of PH and measured the fluorescence lifetimes of individual rotational fine structure levels for the *v'* = 0 level of the $A^{3}\Pi$ state by the high frequency deflection technique; they detected weak predissociations from the $A^{3}\Pi$ state. In 2002, Fitzpatrick et al. (2002) observed the emission spectra of the $A^{3}\Pi$ → $X^{3}\Sigma^{-}$ transition of PH, reported the fluorescence lifetimes of the (1, 0) (2, 0) and (2, 1) bands, and investigated the predissociation dynamics of the $A^{3}\Pi$ state. Later, Fitzpatrick et al. (2003) recorded Sub-Doppler spectra of the $A^{3}\Pi$ → $X^{3}\Sigma^{-}$ transition of PH and reported measurements of the hyperfine coupling constants of the $A^{3}\Pi$ state.

Theoretically, in 1987, Gustafsson et al. (1987) performed *ab initio* calculations on NH using the complete active space self-consistent field (CASSCF) method, and reported the radiative lifetimes of various rovibrational levels in the $A^{3}\Pi$ state. In 2007, Owono et al. (2007) calculated the potential energy curves (PECs), spectroscopic constants and dipole moment functions for the excited and Rydberg states of NH with the internally contracted multireference configuration interaction (icMRCI) approach. Subsequently, Owono et al. (2008) computed various radiative characteristics for the $A^{3}\Pi$ → $X^{3}\Sigma^{-}$ transition of NH including Einstein coefficients, radiative lifetimes and oscillator strengths at the MRCI level. In 2016, Song et al. (2016) obtained the PECs of the twelve Λ-S states and corresponding Ω states for NH using the icMRCI approach including the Davidson correction (+Q). They also calculated the allowed transition dipole moments of four transitions and the lifetimes of the corresponding vibrational levels. On the other hand, seven low-lying Λ-S states of PH were calculated at the MRCI level by Bruna et al. (1981) in 1981; they supposed that the Σ−5 repulsive state was responsible for the predissociation of the $A^{3}\Pi$ state. In 1992, the transition moments of the $A^{3}\Pi$ → $X^{3}\Sigma^{-}$ transition and dipole moments of the first five low-lying states of PH were computed by an *ab initio* effective valence shell Hamiltonian method (Park and Sun, 1992). In 2014, Gao and Gao (2014) investigated the spectroscopic properties of six low-lying Λ-S states and predissociation mechanisms of the $A^{3}\Pi$ state for PH using the icMRCI + Q method.

Molecular laser cooling is achieved by a continuous scattering of a large number of photons, with each cycle of absorption and emission slowing down its translational motion by a small amount. In each cooling cycle, molecules are excited to their higher electronic state, and then return to the initial ground state through spontaneous emission. Photons are emitted in random directions with a symmetric average distribution, so their contribution to the molecule’s momentum averages to zero. Consequently, a molecule is slowed using the transfer of momentum that occurs when it absorbs a colliding photon. The emission in a molecule may populate different vibrational levels, and thus additional repump lasers must be used to bring the population back to continue the photon cycling.

So far, there have not been theoretical investigations reported on laser cooling of PH to the best of our knowledge. Very recently, the $A^{3}\Pi_{1}$ → $X^{3}\Sigma^{-}$ transition of NH has been used to establish a laser cooling scheme based on the *ab initio* calculation by Yan et al. (2021), however, the spin-orbit coupling (SOC) effects on the PECs and vibrational structures were not considered, and the influences of higher electronic states and the spin-orbit splitting of the $X^{3}\Sigma^{-}$ state were not studied. In the present work, by means of highly accurate *ab initio* and dynamical calculations with the SOC effects included, two excellent ultracold molecular candidates from group VA hydrides are identified, which satisfy all known criteria of molecular laser cooling. The paper is organized as follows. The theoretical methods and computational details are briefly described in section 2. In section 3, we present the calculational results, outline the effects of the extra electronic states on laser cooling, and construct two feasible schemes for promising ultracold molecular candidates from group VA hydrides. The conclusions are given in section 4.

## Methods and Computational Details

In the present work, all the *ab initio* calculations of NH and PH are performed in the C_2*v*_ point group using the MOLPRO 2012.1 program package (Werner et al., 2012). The energies of six Λ-S states of NH and PH are calculated using the CASSCF (Werner and Knowles, 1985) method followed by the icMRCI + Q (Langhoff and Davidson, 1974; Knowles and Werner, 1988; Werner and Knowles, 1988) method.

Choosing a proper active space is crucial in the CASSCF and MRCI + Q calculations (Liu et al., 2009; Yu and Bian, 2011; Yu and Bian, 2012). The full valence space is inadequate from our test calculations, thus we add additional orbitals into active space for NH and PH. The inner shell orbitals are included to account for the core-valence correlation effects, and the outer virtual orbitals are involved to give a better description on the dissociation behavior as well as Rydberg character, especially for excited electronic states (Shen et al., 2017). The best balance accuracy and computational performance is to distribute the eight electrons in ten active orbitals corresponding to N 1s2s2p3s3p and H 1s, and we use the aug-cc-pV6Z basis sets for N and H (Dunning and Peterson, 2000; van Mourik et al., 2000). The active space of PH is denoted as CAS (6e, 7o) including the P 3s3p3d_π_ and H 1s orbitals, and the aug-cc-pV6Z basis sets are used for P and H. In the SOC computations, the SOC effect was included by the state interaction approach with the Breit-Pauli Hamiltonian (H_BP_) (Berning et al., 2000), and the SO eigenstates were obtained by diagonalizing *Ĥ* ^*el*^ + *Ĥ* ^*SO*^ in a basis of eigenfunctions of *Ĥ* ^*el*^. Moreover, the *Ĥ* ^*el*^ matrix elements are obtained from the icMRCI + Q calculations, and the *Ĥ* ^*SO*^ matrix elements are acquired from the icMRCI + Q waves functions.

The Einstein spontaneous emission coefficient $A_{\nu^{\prime },J^{\prime }\to \nu ,J}$ from the initial-state (*ν*′, *J′*) to the final-state (*ν*, *J*) is defined by the following expression (Herzberg, 1950):

$$A_{\nu^{\prime },J^{\prime }\to \nu ,J}=3.1361861\times 10^{-7}\frac{S\left(J^{\prime },J\right)}{2J^{\prime }+1}v^{3}{\left|\Psi_{\nu^{\prime },J^{\prime }}\left|M\left(r\right)\right|\Psi_{\nu ,J}\right|}^{2}$$ (1)

where $A_{\nu^{\prime },J^{\prime }\to \nu ,J}$ is in s^−1^ unit, $S\left(J^{\prime },J\right)$ is the Hönl-London rotational intensity factor, *v* is emission frequency in cm^−1^ unit, *M* (*r*) is the transition dipole function in Debye unit, $\Psi_{\nu ,J}$ and $\Psi_{\nu^{\prime },J^{\prime }}$ are the unit normalized radial wave functions.

For a given vibrational level *ν*′, the radiative lifetime $\left(\tau_{\nu^{\prime }}\right)$ is obtained by the following expression:

$$\tau_{\nu^{\prime }}=\frac{1}{\sum_{v}A_{v^{\prime }v}}$$ (2)

The spectroscopic constants of NH and PH, including the adiabatic relative electronic energy referred to the ground state (*T*_*e*_), equilibrium interatomic distance (*R*_*e*_), dissociation energy (*D*_*e*_), the rotational constant (*B*_*e*_), the harmonic and anharmonic vibrational constants (*ω*_*e*_ and *ω*_*e*_ *χ*_*e*_) are determined by solving the nuclear Schrӧdinger equation using the LEVEL 8.0 program (Le Roy, 2007).

## Results and Discussion

### PECs and Molecular Spectroscopic Constants

**FIGURE 1:**
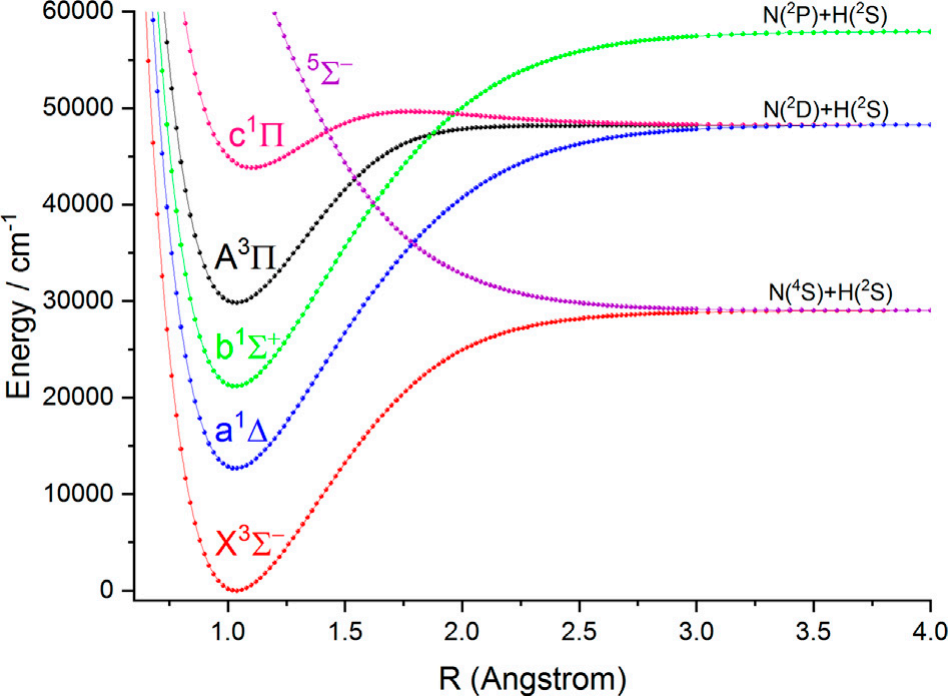
Potential energy curves of NH as a function of the interatomic distance (R) for the six Λ-S states at the icMRCI(8e, 10o)+Q/aug-cc-pV6Z level.

**FIGURE 2:**
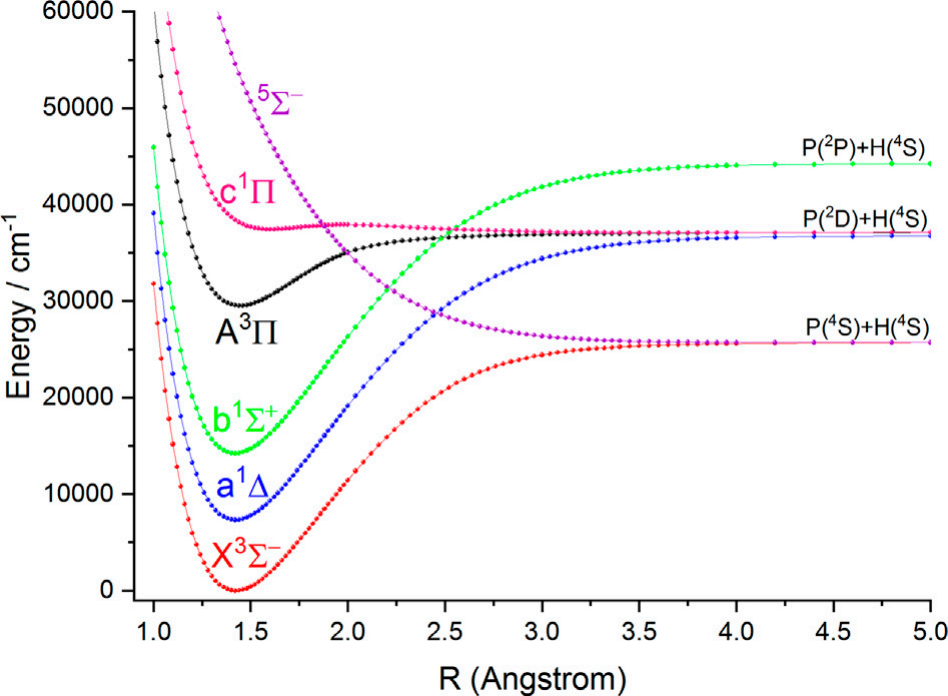
Potential energy curves of PH as a function of the interatomic distance (R) for the six Λ-S states at the icMRCI (6e, 7o) + Q/aug-cc-pV6Z level.

In this work, the PECs of six Λ-S electronic states of NH and PH are computed with the icMRCI + Q method. The first three low-lying electronic states ( $X^{3}\Sigma^{-}$ , $a^{1}\Delta$ and $b^{1}\Sigma^{+}$ ) of NH and PH have the same electronic configuration σ^2^π^2^. The electronic configurations of the excited states $A^{3}\Pi$ and $c^{1}\Pi$ are σ^1^π^3^, which could be considered as involving a pσ → pπ transition within the N/P atom. The electronic configuration of the repulsive state Σ−5 is σ^1^π^2^σ^∗^. The PECs of six Λ-S electronic states of NH and PH are depicted in Figures 1 and 2, respectively. As seen in Figures 1 and 2, the $X^{3}\Sigma^{-}$ and Σ−5 states of NH and PH correlate to the lowest neutral atomic N/PH (S4) + H(S2) limit, the $a^{1}\Delta$ , $A^{3}\Pi$ and $c^{1}\Pi$ states correlate adiabatically to the N/PH (D2)+H(S2) limit, and the $b^{1}\Sigma^{+}$ state corresponds to the N/PH (P2)+H(S2) limit. Since the spectroscopic constants of the $X^{3}\Sigma^{-}$ and $A^{3}\Pi$ states have been measured in experiment for NH and PH, comparing with the available experimental measurements could give an indicator of the accuracy and reliability of our computations. Our calculated spectroscopic constants of five Λ-S states for NH and PH are tabulated in Tables 1 and 2, respectively, comparing with previous experimental and theoretical values.

**TABLE 1:** Spectroscopic constants of the five Λ-S states for NH.

State	Method	*T*_*e*_ (cm^−1^)	*R*_*e*_ (Å)	*ω*_*e*_ (cm^−1^)	*ω*_*e*_ *χ*_*e*_ (cm^−1^)	*D*_*e*_ (eV)	*B*_*e*_ (cm^−1^)
$X^{3}\Sigma^{-}$	This work	0	1.035	3,283.98	82.46	3.6091	16.41
	Expt. ^a^	0	1.0362	3,282.27	78.35	3.601	16.699
	Expt. ^b^	0	1.0372	3,266	78.50		16.67
	Calc. ^c^	0	1.0375	3,292.07	86.66	3.6146	16.74
$a^{1}\Delta$	This work	12,537.40	1.034	3,191.72	68.05	4.4340	16.47
	Expt, ^a^	12,566	1.0341	3,188	68.00		16.439
	Calc, ^c^	12,529.37	1.0341	3,336.04	68.18	4.4209	16.63
$b^{1}\Sigma^{+}$	This work	21,216.85	1.034	3,354.35	78.65	4.5681	16.49
	Expt. ^a^	21,202	1.036	3,352.4	74.24	4.5483	16.705
	Calc. ^c^	21,196.42	1.0322	3,371.33	76.12	4.5534	16.87
$A^{3}\Pi$	This Work	29,824.42	1.036	3,234.88	98.68	2.2989	16.40
	Expt. ^a^	29,807.4	1.037	3,231.2	98.60	2.2875	16.674
	Expt. ^b^	29,818.01	1.0361	3,188			16.69
	Calc. ^c^	29,794.77	1.0368	3,263.32	97.73	2.2803	16.69
$c^{1}\Pi$	This work	43,783.62	1.10	2,124.40		0.7286	14.79
	Expt. ^a^	43,744	1.1106	2,122.64		0.7126	14.537
	Calc. ^c^	43,468.49	1.09	2074.44		0.7442	14.72

a Reference (Huber and Herzberg, 1979).

b Reference (Lents, 1973).

c Reference (Song et al., 2016).

**TABLE 2:** Spectroscopic constants of the five Λ-S states for PH.

State	Method	*T*_*e*_ (cm^−1^)	*R*_*e*_ (Å)	*ω*_*e*_ (cm^−1^)	*ω*_*e*_ *χ*_*e*_ (cm^−1^)	*D*_*e*_ (eV)	*B*_*e*_ (cm^−1^)
$X^{3}\Sigma^{-}$	This work	0	1.422	2,389.89	46.88	3.1890	8.5256
	Expt. ^a^	0	1.4223	2,365.2	44.5		8.5371
	Expt. ^b^	0	1.4221	2,365.2		3.8931	8.537
	Calc. ^c^	0	1.420	2,392.51	47.5	3.18	8.5335
$a^{1}\Delta$	This work	7,326.99	1.422	2,391.75	41.48	3.6511	8.5476
	Expt. ^a^	7,660	1.4302				8.443
	Calc. ^c^	7,140	1.422	2,390.2	42.5	3.65	8.5348
$b^{1}\Sigma^{+}$	This work	14,223.05	1.420	2,408.88	41.15	3.7250	8.5679
	Expt. ^d^	14,325.5 ± 0.1	1.4178 ± 0.0004	2,403.0 ± 0.1	42.0 ± 0.1		8.587 ± 0.003
	Calc. ^c^	14,160.5	1.420	2,409.9	42.3	3.73	8.5668
$A^{3}\Pi$	This work	29,528.42	1.445	2,127.89	148.10	0.9441	8.2883
	Expt. ^b^	29,484	1.4458	2030.6	98.5		8.259
	Calc. ^c^	29,348.15	1.448	2,237.6	167.6	0.92	8.2539
$c^{1}\Pi$	This work	37,452.45					
	Expt. ^e^	37,500					
	Calc. ^c^	37,267					

a Reference (Huber and Herzberg, 1979).

b Estimated using isotope relations in Reference (Rostas et al., 1974).

c Reference (Gao and Gao, 2014).

d Reference (Droege and Engelking, 1984).

e Reference (Di Stefano et al., 1978).

As seen in Table 1, for the ground state $X^{3}\Sigma^{-}$ of NH, our computed *R*_*e*_, *ω*_*e*_ and *ω*_*e*_ *χ*_*e*_ values (1.035 Å, 3,283.98 and 82.46 cm^−1^) reproduce the experimental data (1.0362 Å, 3,282.27 and 78.35 cm^−1^) very well (Huber and Herzberg, 1979). It is also encouraging to see that our calculated *D*_*e*_ value of 3.6091 eV for the $X^{3}\Sigma^{-}$ state of NH is in excellent agreement with the experimental result of 3.601 eV (Huber and Herzberg, 1979). Concerning the first excited state $a^{1}\Delta$ of NH, our computed *T*_*e*_, *ω*_*e*_ and *ω*_*e*_ *χ*_*e*_ values are 12,537.40, 3,191.72 and 68.05 cm^−1^, respectively, which are in excellent accordance with the experimental data (12,566, 3,188 and 68.00 cm^−1^) (Huber and Herzberg, 1979) and much improved compared with the previous calculations (12,529.37, 3,336.04 and 68.18 cm^−1^) (Song et al., 2016). The calculated *R*_*e*_ and *B*_*e*_ values (1.034 Å and 16.47 cm^−1^) of the $a^{1}\Delta$ state are in excellent accordance with the measurements (1.0341 Å and 16.439 cm^−1^) (Huber and Herzberg, 1979). Next in energy is the $b^{1}\Sigma^{+}$ state of NH. Our calculated *T*_*e*_ value of the $b^{1}\Sigma^{+}$ state (21,216.85 cm^−1^) is in excellent agreement with the experimental data (21,202 cm^−1^) (Huber and Herzberg, 1979) and theoretical value (21,196.42 cm^−1^) (Song et al., 2016). The *R*_*e*_ and *ω*_*e*_ values of the $b^{1}\Sigma^{+}$ state computed by us (1.034 Å and 3,354.35 cm^−1^) are much closer to the experimental results (1.036 Å and 3352.4 cm^−1^) compared with the previous theoretical values (1.0322 Å and 3,371.33 cm^−1^). Besides, our computed *ω*_*e*_ *χ*_*e*_, *D*_*e*_ and *B*_*e*_ values of the $b^{1}\Sigma^{+}$ state agree well with the experimental results. The experimental *T*_*e*_ value of the $A^{3}\Pi$ state of NH is (29,818.01 cm^−1^) (Lents, 1973), whereas our calculated *T*_*e*_ value is 29,824.42 cm^−1^, which is better than the previous computational value (29,794.77 cm^−1^) (Song et al., 2016). The *R*_*e*_, *ω*_*e*_, *ω*_*e*_ *χ*_*e*_ and *D*_*e*_ values of the $A^{3}\Pi$ state computed by us (1.036 Å, 3,234.88 cm^−1^, 98.68 cm^−1^ and 2.2989 eV) agree very well with the corresponding experimental data (1.037 Å, 3,231.2 cm^−1^, 98.60 cm^−1^ and 2.2875 eV) (Huber and Herzberg, 1979). For the $c^{1}\Pi$ state of NH, the excitation energy is calculated to be 43,783.62 cm^−1^, noticeably higher than that obtained in the previous calculations (43,468.49 cm^−1^) (Song et al., 2016), and thus in much better agreement with the measured value of 43,744 cm^−1^(Huber and Herzberg, 1979). The calculated *R*_*e*_, *ω*_*e*_ and *D*_*e*_ values of the $c^{1}\Pi$ state of NH are 1.10 Å, 2,124.40 cm^−1^ and 0.7286 eV, respectively, which agree excellently with the experimental results (1.1106 Å, 2,122.64 cm^−1^ and 0.7126 eV). In Figure 1, for the $c^{1}\Pi$ state of NH, the bump of the PEC may result from an avoided crossing between the $c^{1}\Pi$ state and a higher Π1 state. The resultant potential barrier is 1,293.26 cm^−1^ at 1.80 Å relative to the dissociation limit in this work, which is in very good agreement with the value of 1,292.12 cm^−1^ calculated by Song et al. (2016)

In Table 2, our calculated *R*_*e*_ and *B*_*e*_ values of the $X^{3}\Sigma^{-}$ state of PH are 1.422 Å and 8.5256 cm^−1^, respectively, which agree perfectly with the experimental measurements (1.4223 Å and 8.5371 cm^−1^) (Huber and Herzberg, 1979). The present calculated *ω*_*e*_ and *ω*_*e*_ *χ*_*e*_ values of the $X^{3}\Sigma^{-}$ state are 2,389.89 cm^−1^ and 46.88 cm^−1^, respectively, which are in very good agreement with the previous theoretical results (2,392.51 cm^−1^ and 47.5 cm^−1^) (Gao and Gao, 2014). For the $a^{1}\Delta$ state of PH, our calculated *T*_*e*_ value (7,326.99 cm^−1^) is much closer to the experimental value (7,660 cm^−1^) (Huber and Herzberg, 1979) than the old one (7,140 cm^−1^) (Gao and Gao, 2014). The *R*_*e*_, *ω*_*e*_, *ω*_*e*_ *χ*_*e*_, *D*_*e*_ and *B*_*e*_ values of the $a^{1}\Delta$ state are computed to be 1.422 Å, 2,391.75 cm^−1^, 41.48 cm^−1^, 3.6511 eV and 8.5476 cm^−1^, respectively, which agree very well with the corresponding theoretical results (1.422 Å, 2,390.2 cm^−1^, 42.5 cm^−1^, 3.65 eV and 8.5348 cm^−1^) (Gao and Gao, 2014). The excitation energy of the present work for the $b^{1}\Sigma^{+}$ state of PH is computed to be 14,223.05 cm^−1^, which is much closer to the experimental result of 14,325.5 ± 0.1 cm^−1^ (Droege and Engelking, 1984) than the previous calculation (14,160.5 cm^−1^) (Gao and Gao, 2014). It is also encouraging to see that the present values of *R*_*e*_ and *ω*_*e*_ values for the $b^{1}\Sigma^{+}$ state are 1.420 Å and 2,408.88 cm^−1^, respectively, which are in excellent agreement with those derived experimentally, 1.4178 ± 0.0004 Å and 2,403.0 ± 0.1 cm^−1^ (Droege and Engelking, 1984). In addition, the calculated value (41.15 cm^−1^) for *ω*_*e*_ *χ*_*e*_ of the $b^{1}\Sigma^{+}$ state agrees very well with the experimental value of 42.0 ± 0.1 cm^−1^ (Droege and Engelking, 1984). Besides, the computed *D*_*e*_ and *B*_*e*_ values of the $b^{1}\Sigma^{+}$ state (3.7250 eV and 8.5679 cm^−1^) are in very good agreement with the theoretical results (3.73 eV and 8.5668 cm^−1^) (Gao and Gao, 2014). The experimental excitation energy to the $A^{3}\Pi$ state of PH is 29,484 cm^−1^ (Rostas et al., 1974), while the present value is 29,528.42 cm^−1^, which is much improved compared with the previous theoretical value 29,348.15 cm^−1^ (Gao and Gao, 2014). For the $A^{3}\Pi$ state, the agreement between our computed *R*_*e*_, *D*_*e*_ and *B*_*e*_ values (1.445 Å, 0.9441 eV and 8.2883 cm^−1^) and the theoretical data (1.448 Å, 0.92 eV and 8.2539 cm^−1^) (Gao and Gao, 2014) is very good. There are some deviations between the calculational and experimental (Rostas et al., 1974) results for the *ω*_*e*_ and *ω*_*e*_ *χ*_*e*_ values of the $A^{3}\Pi$ state, although the experimental values were estimated based on the isotopic relation, and may have large uncertainties (Rostas et al., 1974). The experimental *T*_*e*_ value of the $c^{1}\Pi$ state of PH is 37,500 cm^−1^(Di Stefano et al., 1978), whereas our calculated *T*_*e*_ value is 37,452.45 cm^−1^, which is much better than the previous computational value of 37,267 cm^−1^. (Gao and Gao, 2014).

**FIGURE 3:**
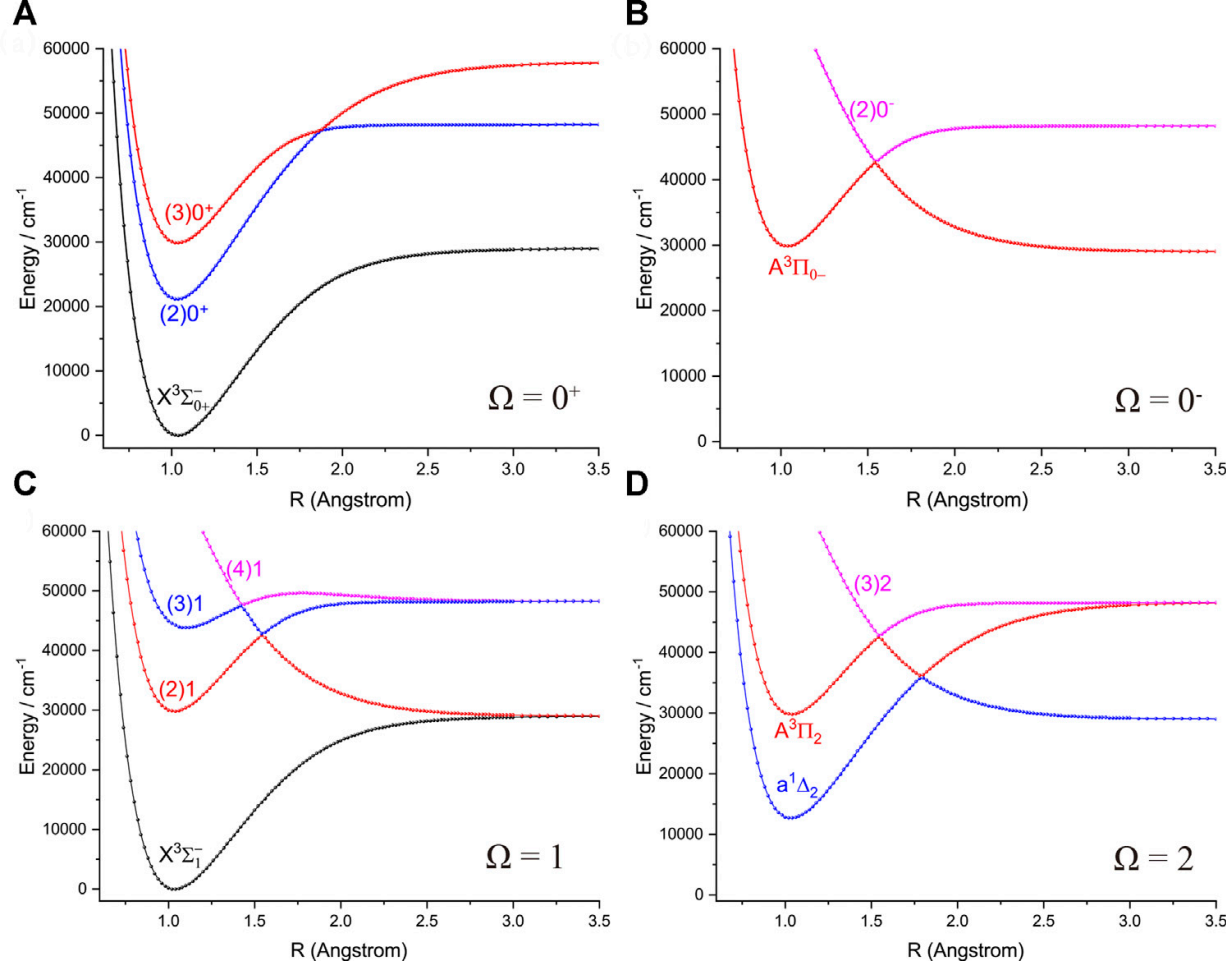
Potential energy curves of NH as a function of the interatomic distance (R) for **(A)** Ω = 0^+^, **(B)** Ω = 0^−^, **(C)** Ω = 1 and **(D)** Ω = 2 at the icMRCI + Q level.

**FIGURE 4:**
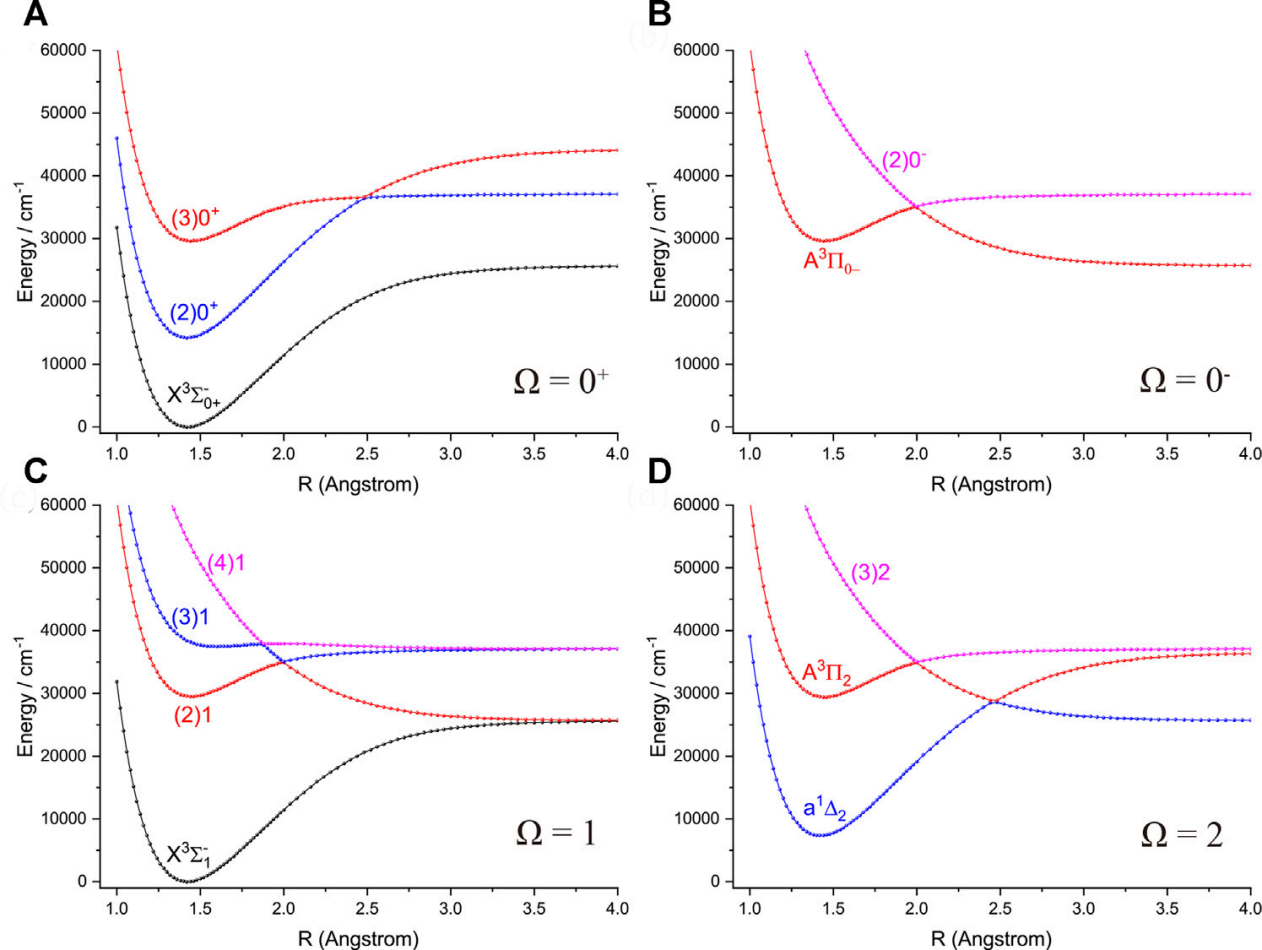
Potential energy curves of PH as a function of the interatomic distance (R) for **(A)** Ω = 0^+^, **(B)** Ω = 0^−^, **(C)** Ω = 1 and **(D)** Ω = 2 at the icMRCI + Q level.

The six Λ-S states $X^{3}\Sigma^{-}$ , $a^{1}\Delta$ , $b^{1}\Sigma^{+}$ , $A^{3}\Pi$ , $c^{1}\Pi$ and Σ−5 of NH and PH split into 12 Ω states when the SOC effects are taken into account, including three states with Ω = $0^{+}$ ( $X^{3}\Sigma_{0+}^{-}$ , $b^{1}\Sigma_{0+}^{+}$ and $A^{3}\Pi_{0+}$ ), two states with Ω = $0^{-}$ ( $A^{3}\Pi_{0-}$ and ^5^ $\Sigma_{0-}^{-}$ ), four states with Ω = 1 ( $X^{3}\Sigma_{1}^{-}$ , $A^{3}\Pi_{1}$ , $c^{1}\Pi_{1}$ and ^5^ $\Sigma_{1}^{-}$ ), and three states with Ω = 2 ( $a^{1}\Delta_{2}$ , $A^{3}\Pi_{2}$ and ^5^ $\Sigma_{2}^{-}$ ). The PECs of 12 Ω states of NH and PH are depicted in Figures 3 and 4, respectively. The spectroscopic constants of the 9 Ω states of NH and PH including the $X^{3}\Sigma_{0+}^{-}$ , $X^{3}\Sigma_{1}^{-}$ , $a^{1}\Delta_{2}$ , $b^{1}\Sigma_{0+}^{+}$ , $A^{3}\Pi_{0+}$ , $A^{3}\Pi_{0-}$ , $A^{3}\Pi_{1}$ , $A^{3}\Pi_{2}$ and $c^{1}\Pi_{1}$ states are displayed in Tables 3 and 4, respectively. As seen in Table 3, the spectroscopic constants *T*_*e*_, *R*_*e*_, *ω*_*e*_, *ω*_*e*_ *χ*_*e*_, *D*_*e*_ and *B*_*e*_ values of the four Λ-S states $X^{3}\Sigma^{-}$ , $a^{1}\Delta$ , $b^{1}\Sigma^{+}$ and $c^{1}\Pi$ of NH are nearly same to those of the corresponding Ω states. For the four Λ-S states of NH, the energy difference between the four Λ-S states and the corresponding Ω states is less than 1 cm^−1^. While the SO splitting values of the $A^{3}\Pi_{1}-A^{3}\Pi_{2}$ , $A^{3}\Pi_{0-}-A^{3}\Pi_{1}$ and $A^{3}\Pi_{0+}-A^{3}\Pi_{0-}$ states are 34.04, 34.22 and 0.17 cm^−1^, respectively, which are in excellent accordance with the computational values (the splitting values of the $A^{3}\Pi_{1}-A^{3}\Pi_{2}$ and $A^{3}\Pi_{0-}-A^{3}\Pi_{1}$ states are 34.06 and 34.00 cm^−1^, respectively) (Yan et al., 2021). In Table 4, the energy difference between the four Λ-S states ( $X^{3}\Sigma^{-}$ , $a^{1}\Delta$ , $b^{1}\Sigma^{+}$ and $c^{1}\Pi$ ) and the corresponding Ω states of PH is less than 6 cm^−1^, whereas the SO splitting values of the $A^{3}\Pi_{1}-A^{3}\Pi_{2}$ , $A^{3}\Pi_{0-}-A^{3}\Pi_{1}$ and $A^{3}\Pi_{0+}-A^{3}\Pi_{0-}$ states are 100.32, 102.83 and 1.16 cm^−1^, respectively. In view of the above, the SOC effects should be taken into account for the spectroscopic study of excited states for NH and PH and thus are important for laser cooling of NH and PH.

**TABLE 3:** Spectroscopic constants of the 9 Ω states for NH.

State	Method	*T*_*e*_ (cm^−1^)	*R*_*e*_ (Å)	*ω*_*e*_ (cm^−1^)	*ω*_*e*_ *χ*_*e*_ (cm^−1^)	*D*_*e*_ (eV)	*B*_*e*_ (cm^−1^)
$X^{3}\Sigma_{0+}^{-}$	This work	0	1.035	3,283.63	82.68	3.6091	16.41
	Calc^a^	0	1.0375	3,292.26		3.6148	
$X^{3}\Sigma_{1}^{-}$	This work	0.22	1.035	3,283.54	82.40	3.6091	16.41
	Calc. ^a^	0.02	1.0375	3,292.27		3.6149	
$a^{1}\Delta_{2}$	This work	12,537.58	1.034	3,191.63	68.04	4.4222	16.47
	Calc. ^a^	12,529.45	1.0343	3,335.26		4.4213	
$b^{1}\Sigma_{0+}^{+}$	This work	21,216.79	1.032	3,354.90	78.61	4.5682	16.49
	Calc. ^a^	21,196.78	1.0321	3,372.28		4.5536	
$A^{3}\Pi_{2}$	This work	29,790.58	1.036	3,234.68	98.61	2.3029	16.40
	Calc. ^a^	29,794.95	1.0379	3,265.69		2.2827	
	Calc. ^b^	29,960	1.0364	3,215.71	91.4		16.623
$A^{3}\Pi_{1}$	This work	29,824.62	1.036	3,234.56	98.63	2.2992	16.40
	Calc. ^a^	29,800.03	1.0378	3,266.32		2.2819	
	Calc. ^b^	29,925	1.0364	3,215.56	91.5		16.621
$A^{3}{\text{Π}}_{0-}$	This work	29,858.84	1.036	3,234.11	98.62	2.2950	16.40
	Calc. ^a^	29,805.23	1.0317	3,266.31		2.2824	
$A^{3}\Pi_{0+}$	This work	29,859.01	1.036	3,234.02	98.62	2.2944	16.40
	Calc. ^a^	29,805.89	1.0316	3,265.45		2.2782	
*c* ^1^Π_1_	This work	43,783.98	1.10	2,124.29		0.6788	15.01
	Calc. ^a^	43,466.27	1.0983	2073.57		0.7442	

a Reference (Song et al., 2016).

b Reference (Yan et al., 2021).

**TABLE 4:** Spectroscopic constants of the 9 Ω states for PH.

State	Method	*T*_*e*_ (cm^−1^)	*R*_*e*_ (Å)	*ω*_*e*_ (cm^−1^)	*ω*_*e*_ *χ*_*e*_ (cm^−1^)	*D*_*e*_ (eV)	*B*_*e*_ (cm^−1^)
$X^{3}\Sigma_{0+}^{-}$	This work	0	1.4220	2,395.42	47.75	3.1892	8.5257
	Calc.^a^	0	1.4238	2,385.05	47.68		8.5197
$X^{3}\Sigma_{1}^{-}$	This work	3.09	1.4220	2,395.39	47.74	3.1891	8.5257
	Calc.^a^	3.0	1.4238	2,385.07	47.68		8.5197
$a^{1}\Delta_{2}$	This work	7,329.88	1.422	2,394.36	42.82	3.6252	8.5477
	Calc.^a^	7,665.2	1.4227	2,386.49	42.93		8.5323
$b^{1}\Sigma_{0+}^{+}$	This work	14,228.96	1.420	2,409.75	42.62	3.7253	8.5677
	Calc.^a^	14,340.8	1.4202	2,405.84	42.64		8.5626
$A^{3}\Pi_{2}$	This work	29,430.34	1.445	2,137.87	147.66	0.9565	8.2882
$A^{3}\Pi_{1}$	This work	29,530.66	1.445	2,128.39	148.13	0.9444	8.2886
$A^{3}\Pi_{0-}$	This work	29,633.49	1.445	2,118.55	148.66	0.9317	8.2891
$A^{3}\Pi_{0+}$	This work	29,634.65	1.445	2,120.72	148.93	0.9315	8.2892
*c* ^1^Π_1_	This work	37,457.56					

a Reference (Gao and Gao, 2014).

Accurate determination of *T*_*e*_ is very important for evaluating the pump and repump wavelengths in laser-cooling cycles, and our computed *T*_*e*_ values, which agree very well with the corresponding experimental ones, give us confidence in the subsequent investigation on molecular laser cooling of NH and PH.

### The Effects of the Extra Electronic States on Laser Cooling

**FIGURE 5:**
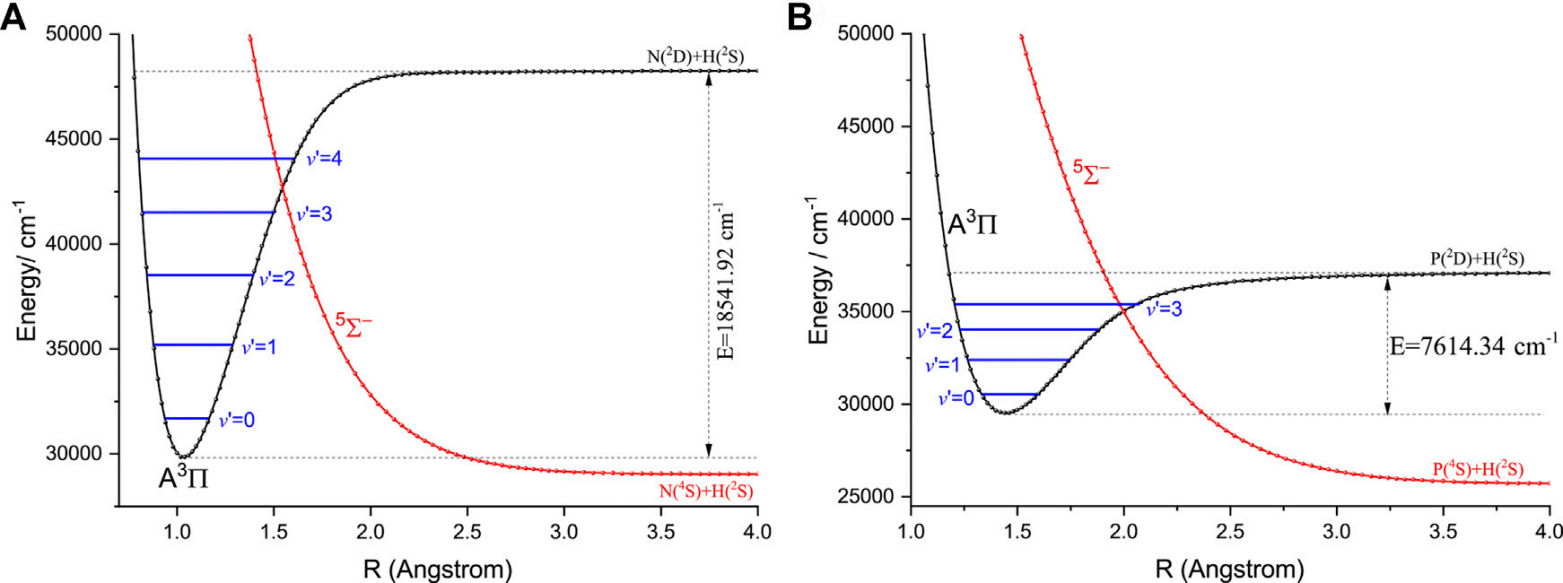
An amplified view of crossing regions of the $A^{3}\Pi$ and ${\;}^{5}\Sigma^{-}$ potential energy curves for NH **(A)** and PH **(B)** as a function of the interatomic distance (R).

Here, we discuss the effects of the extra electronic states on direct laser cooling of NH and PH. An amplified view of crossing regions of PECs of the $A^{3}\Pi$ and Σ−5 states for NH and PH is depicted in Figure 5. We can see that the dissociation energies of the $A^{3}\Pi$ state of NH and PH are 18,541.92 and 7,614.34 (Di Stefano et al., 1978)cm^−1^, respectively. The $A^{3}\Pi$ and Σ−5 states of NH and PH have a crossing point, which can lead to nonradiative transition (Wu et al., 2019), and may cause predissociation. In the polyatomic molecule cases, this kind of electronic state crossing in a diatomic molecule will become potential energy surface intersections including multiple electronic states (Liu et al., 2003; Zhao et al., 2006). We find that the locations of crossing point between the $A^{3}\Pi$ and Σ−5 states of NH and PH are higher than the corresponding *ν*′ = 2 vibrational levels of the $A^{3}\Pi$ state (4,163 and 989 cm^−1^, respectively) indicating that the crossings with higher electronic states would not affect laser cooling. The large $f_{00}$ values of the $A^{3}\Pi_{2}$ → $X^{3}\Sigma_{1}^{-}$ transition for NH and PH (NH: 0.9994; PH: 0.9675) suggest that the two molecules are promising candidates for efficient and rapid laser cooling. This conclusion can be backed up by experimentalists, since the (1, 1) band of the $A^{3}\Pi$ → $X^{3}\Sigma^{-}$ transition for NH and PH has been observed (Funke, 1935; Fitzpatrick et al., 2002). Generally speaking, a larger atomic mass difference for the diatomic candidate is desirable by experimentalists, and in this respect, PH is a better laser cooling candidate than NH.

It should be noted that the transitions between the singlet and triplet states are allowed when the SOC effects are considered. The effects of the intermediate electronic states $\left(a^{1}\Delta_{2}\;\text{and}\;b^{1}\Sigma_{0+}^{+}\right)$ of NH and PH on laser cooling are discussed below. There are two intermediate electronic states $a^{1}\Delta_{2}$ and $b^{1}\Sigma_{0+}^{+}$ in the constructed laser cooling schemes for NH/PH based on the $A^{3}\Pi_{2}$ → $X^{3}\Sigma_{1}^{-}$ transition, where NH/PH molecules are excited from the $X^{3}\Sigma_{1}^{-}$ (*v* = 0) state to the $A^{3}\Pi_{2}$ (*v*′ = 0) state, then they may decay to the $X^{3}\Sigma_{1}^{-}$ or $a^{1}\Delta_{2}$ state rather than the $b^{1}\Sigma_{0+}^{+}$ state since the $A^{3}\Pi_{2}$ → $b^{1}\Sigma_{0+}^{+}$ transition is forbidden according to the selection rules. So the intermediate electronic state $b^{1}\Sigma_{0+}^{+}$ does not interfere with the laser-cooling. In addition, the absolute transition dipole moments (TDMs) of the $A^{3}\Pi_{2}$ → $a^{1}\Delta_{2}$ transition for NH and PH are shown in Supplementary Figure S1. As seen, the TDMs values of the $A^{3}\Pi_{2}$ → $a^{1}\Delta_{2}$ transition for NH and PH are 0.000495 and 0.000793 debye (0.082% and 0.1169% of the corresponding $A^{3}\Pi_{2}$ → $X^{3}\Sigma_{1}^{-}$ transition) at corresponding *R*_*e*_. The vibrational branching loss ratios $\left(\eta_{i}\right)\left[\eta_{i}=A_{A^{3}\Pi_{2}\to a^{1}\Delta_{2}}/\left(A_{A^{3}\Pi_{2}\left(v^{\prime }=0\right)\to X^{3}\Sigma_{1}^{-}\left(v\right)}+A_{A^{3}\Pi_{2}\to a^{1}\Delta_{2}}\right)\right]$ of the $A^{3}\Pi_{2}$ → $a^{1}\Delta_{2}$ $\left(\eta_{1}\right)$ transition for NH and PH are extremely small (NH: 1.81 × 10^–8^; PH: 1.08 × 10^–6^), and much smaller than the experimental value of YO ( η (YO) < 4 × 10^–4^) (Hummon et al., 2013). The extremely small vibrational branching loss ratios of the $A^{3}\Pi_{2}$ → $a^{1}\Delta_{2}$ transition for NH and PH indicate that the $a^{1}\Delta_{2}$ intermediate electronic state will not interfere with the laser-cooling. Hence, we will construct feasible three-laser cooling schemes for NH and PH on the basis of the $A^{3}\Pi_{2}$ → $X^{3}\Sigma_{1}^{-}$ transition in the next section, which satisfy all known criteria including the fourth one proposed in our recent work (Li et al., 2020).

### Laser Cooling Schemes Proposed for NH and PH Using Specific Spin-Orbit States

Since the SOC effects are important as shown above, we construct the schemes for laser cooling of NH and PH using the spin-orbit states $A^{3}\Pi_{2}$ and $X^{3}\Sigma_{1}^{-}$ . We find that only the $A^{3}\Pi_{2}$ → $X^{3}\Sigma_{1}^{-}$ transition can ensure a closed-loop cooling cycles in the six possible transitions ( $A^{3}\Pi_{2}$ → $X^{3}\Sigma_{1}^{-}$ , $A^{3}\Pi_{1}$ → $X^{3}\Sigma_{1}^{-}$ , $A^{3}\Pi_{1}$ → $X^{3}\Sigma_{0+}^{-}$ , $A^{3}\Pi_{0+}$ → $X^{3}\Sigma_{1}^{-}$ , $A^{3}\Pi_{0+}$ → $X^{3}\Sigma_{0+}^{-}$ and $A^{3}\Pi_{0-}$ → $X^{3}\Sigma_{1}^{-}$ ) from the $A^{3}\Pi_{\Omega }$ . The $A^{3}\Pi_{2}$ → $X^{3}\Sigma_{0+}^{-}$ and $A^{3}\Pi_{0-}$ → $X^{3}\Sigma_{0+}^{-}$ transitions for NH and PH are forbidden according to the selection rules of transitions between the Ω states. In addition, the $A^{3}\Pi_{2}$ state of NH and PH is the energetically lowest-lying state in the 4 Ω states ( $A^{3}\Pi_{0+}$ , $A^{3}\Pi_{0-}$ , $A^{3}\Pi_{1}$ and $A^{3}\Pi_{2}$ ), which can avoid the interference from the other $A^{3}\Pi_{\Omega }$ states ( $A^{3}\Pi_{0+}$ , $A^{3}\Pi_{0-}$ and $A^{3}\Pi_{1}$ ) and ensure a closed-loop cooling cycles. In the constructed laser cooling schemes for NH/PH molecules based on the $A^{3}\Pi_{2}$ → $X^{3}\Sigma_{1}^{-}$ transition, NH/PH molecules are excited from the $X^{3}\Sigma_{1}^{-}$ (*v* = 0) state to the $A^{3}\Pi_{2}$ (*v*′ = 0) state, then they will decay to the $X^{3}\Sigma_{1}^{-}$ state rather than the $X^{3}\Sigma_{0+}^{-}$ state according to the selection rules, and the ultracold NH/PH will be produced through the constructed schemes when the process of cooling cycles repeats constantly. Consequently, the $A^{3}\Pi_{2}$ (*v'*) → $X^{3}\Sigma_{1}^{-}$ (*v*) transition of NH and PH is used to establish corresponding laser cooling schemes in this work.

**FIGURE 6:**
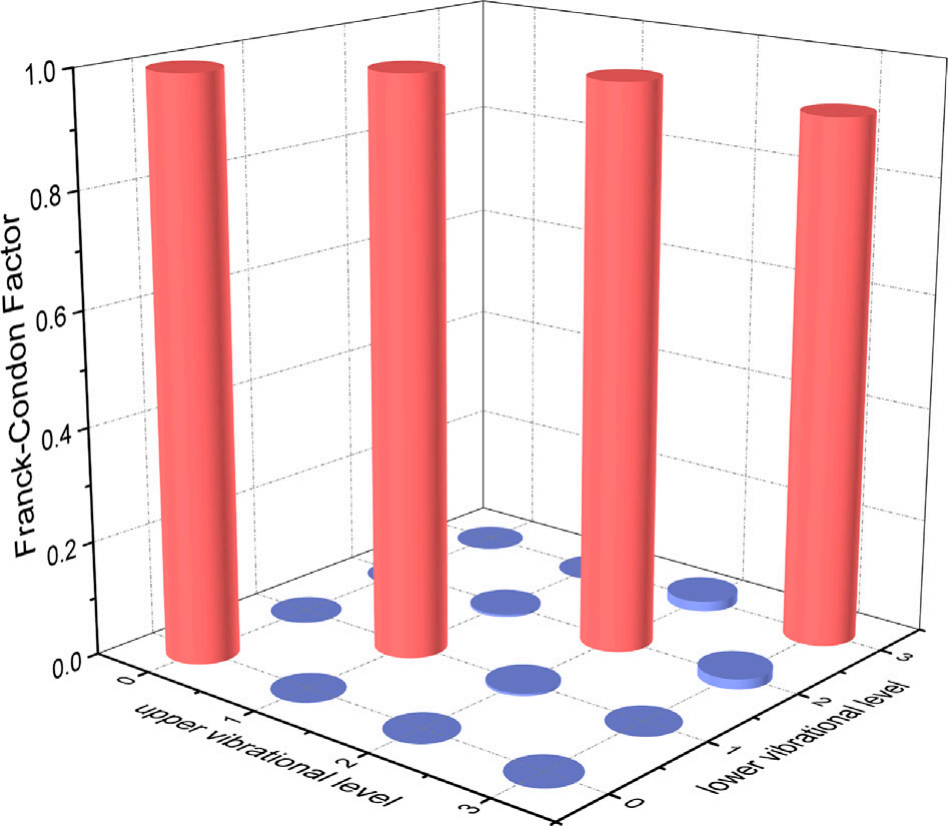
Franck-Condon factors of the $A^{3}\Pi_{2}$ (*v'* ≤ 3) → $X^{3}\Sigma_{1}^{-}$ (*v* ≤ 3) transitions for NH, calculated at the icMRCI + Q level.

**FIGURE 7:**
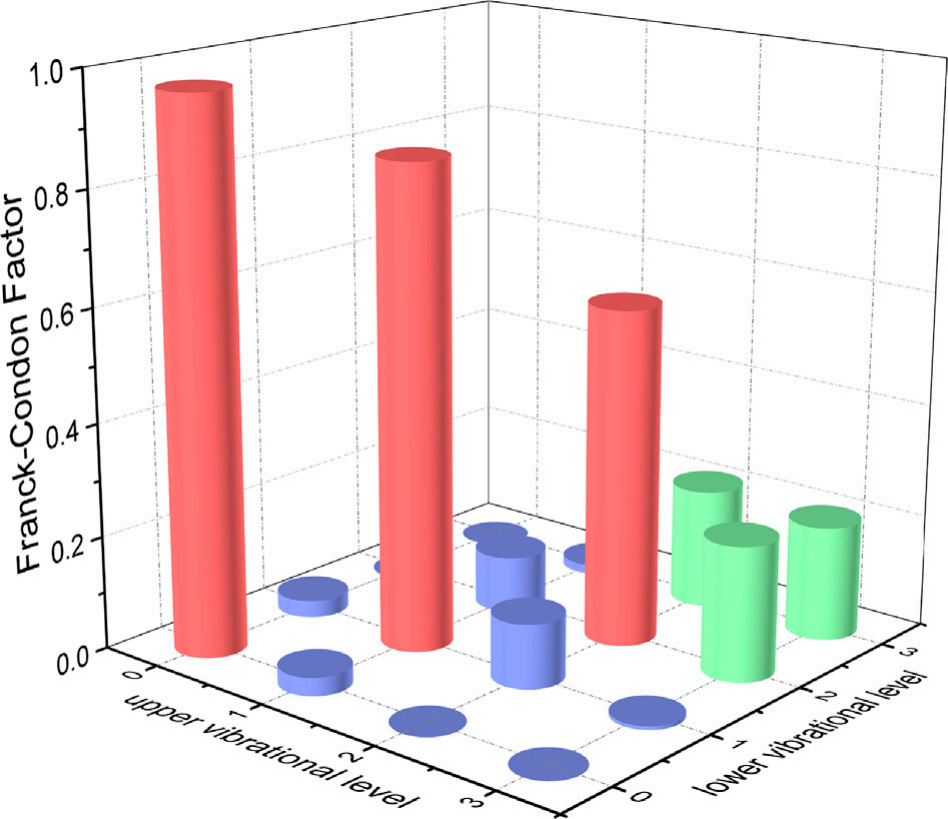
Franck-Condon factors of the $A^{3}\Pi_{2}$ (*v'* ≤ 3) → $X^{3}\Sigma_{1}^{-}$ (*v* ≤ 3) transitions for PH, calculated at the icMRCI + Q level.

The permanent dipole moments (PDMs) and TDMs for the $A^{3}\Pi_{2}$ → $X^{3}\Sigma_{1}^{-}$ transition of NH and PH at the icMRCI + Q level are shown in Supplementary Figure S2. The TDMs of NH and PH decrease with the increasing interatomic distance and are 0.6059 and 0.6788 debye, respectively, at corresponding *R*_*e*_. The FCFs $\left(f_{\nu^{\prime }\nu }\right)$ values of the $A^{3}\Pi_{2}$ → $X^{3}\Sigma_{1}^{-}$ transition for NH and PH are computed and plotted in Figures 6 and 7, respectively. We can clearly see that the $f_{\nu^{\prime }\nu }$ values of Δν=0 vibrational levels of the $A^{3}\Pi_{2}$ → $X^{3}\Sigma_{1}^{-}$ transition for NH and PH are remarkably higher than those for the off-diagonal terms. The $f_{00}$ values of the $A^{3}\Pi_{2}$ → $X^{3}\Sigma_{1}^{-}$ transition for NH (0.9994) and PH (0.9675) are so large that the spontaneous decays to *ν* = 1, 2 vibrational levels of the corresponding $X^{3}\Sigma_{1}^{-}$ state are highly restricted. We will use the *v'* = 0, 1 levels of the corresponding $A^{3}\Pi_{2}$ state of NH and PH with three lasers to establish laser cooling cycles on the basis of the $A^{3}\Pi_{2}$ → $X^{3}\Sigma_{1}^{-}$ transition. Owing to the relative strengths of the photon loss pathways are more directly related to the vibrational branching ratios $R_{\nu^{\prime }\nu }$ than the $f_{\nu^{\prime }\nu }$ in the laser cooling cycle, the Einstein spontaneous emission coefficient $A_{\nu^{\prime }\nu }$ and $R_{\nu^{\prime }\nu }$ of the $A^{3}\Pi_{2}$ → $X^{3}\Sigma_{1}^{-}$ transition for NH and PH are calculated and presented in Tables 5 and 6, respectively. As seen, a very large $A_{00}$ (NH: 2.10×10^6^ s^−1^, PH: 1.90×10^6^ s^−1^) and very low scattering probabilities into off-diagonal bands of NH and PH contribute to a desirable condition for efficient and rapid optical cycles.

**TABLE 5:** Calculated Einstein A coefficients $A_{\nu^{\prime }\nu }$ and vibrational branching ratio $R_{\nu^{\prime }\nu }$ of the $A^{3}\Pi_{2}$ → $X^{3}\Sigma_{1}^{-}$ transition for NH.

	ν' = 0		ν' = 1		ν' = 2		ν' = 3	
	$A_{\nu^{\prime }\nu }$	$R_{\nu^{\prime }\nu }$	$A_{\nu^{\prime }\nu }$	$R_{\nu^{\prime }\nu }$	$A_{\nu^{\prime }\nu }$	$R_{\nu^{\prime }\nu }$	$A_{\nu^{\prime }\nu }$	$R_{\nu^{\prime }\nu }$
ν = 0	2.10 × 10^6^	0.9952	5.05 × 10^4^	3.34 × 10^–2^	4.27 × 10^3^	3.95 × 10^–3^	1.04 × 10^3^	1.39 × 10^–3^
ν = 1	9.57 × 10^3^	4.54 × 10^–3^	1.44 × 10^6^	0.9558	1.16 × 10^5^	1.68 × 10^–1^	1.27 × 10^4^	1.69 × 10^–2^
ν = 2	4.79 × 10^2^	2.27 × 10^–4^	1.53 × 10^4^	1.01 × 10^–2^	9.42 × 10^5^	0.8712	1.86 × 10^5^	0.2478
ν = 3	71	3.37 × 10^–5^	8.06 × 10^2^	5.34 × 10^–4^	1.83 × 10^4^	1.69 × 10^–2^	5.31 × 10^5^	0.7090

**TABLE 6:** Calculated Einstein A coefficients $A_{\nu^{\prime }\nu }$ and vibrational branching ratio $R_{\nu^{\prime }\nu }$ of the $A^{3}\Pi_{2}$ → $X^{3}\Sigma_{1}^{-}$ transition for PH.

	ν' = 0		ν' = 1		ν' = 2		ν' = 3	
	$A_{\nu^{\prime }\nu }$	$R_{\nu^{\prime }\nu }$	$A_{\nu^{\prime }\nu }$	$R_{\nu^{\prime }\nu }$	$A_{\nu^{\prime }\nu }$	$R_{\nu^{\prime }\nu }$	$A_{\nu^{\prime }\nu }$	$R_{\nu^{\prime }\nu }$
ν = 0	1.90 × 10^6^	0.9977	1.88 × 10^5^	0.1195	7.56 × 10^3^	6.05 × 10^–3^	3.81 × 10^1^	4.22 × 10^–5^
ν = 1	3.84 × 10^3^	2.02 × 10^–3^	1.37 × 10^6^	0.8680	4.38 × 10^5^	0.3506	6.16 × 10^4^	6.83 × 10^–2^
ν = 2	4.55 × 10^2^	2.39 × 10^–4^	1.79 × 10^4^	0.0113	7.60 × 10^5^	0.6084	5.79 × 10^5^	0.6426
ν = 3	0.5671	2.98 × 10^–7^	1.78 × 10^3^	1.13 × 10^–3^	3.89 × 10^4^	3.12 × 10^–2^	2.12 × 10^5^	0.2349

The $R_{\nu^{\prime }\nu }$ are assessed using the following expression:

$$R_{\nu^{\prime }\nu }=\frac{A_{\nu^{\prime }\nu }}{\sum_{v}A_{\nu^{\prime }\nu }}$$ (3)

In addition, the Doppler temperatures ( $T_{Doppler}=h/\left(4k_{B}\pi \tau \right)$ , where h is Planck’s constant, *k*_*B*_ is Boltzmann’s constant, and *τ* is the radiative lifetime) of the $A^{3}\Pi_{2}$ (*ν*′ = 0) → $X^{3}\Sigma_{1}^{-}$ (*ν* = 0) transition of NH and PH are 8.06 and 7.27 *µK*, respectively, the radiative lifetimes $\left(\tau_{\nu^{\prime }}\right)$ for main cooling transition of NH and PH are 474 and 526 ns, respectively, and the recoil temperatures $\left(T_{recoil}=h^{2}/\left(mk_{B}\lambda^{2}\right),\;\text{where}\;\text{λ}\;\text{is}\;\text{the}\;\text{laser}\;\text{wavelength}\right)$ for main cooling transition of NH and PH are 1.13 and 5.12 *µK*, respectively.

**FIGURE 8:**
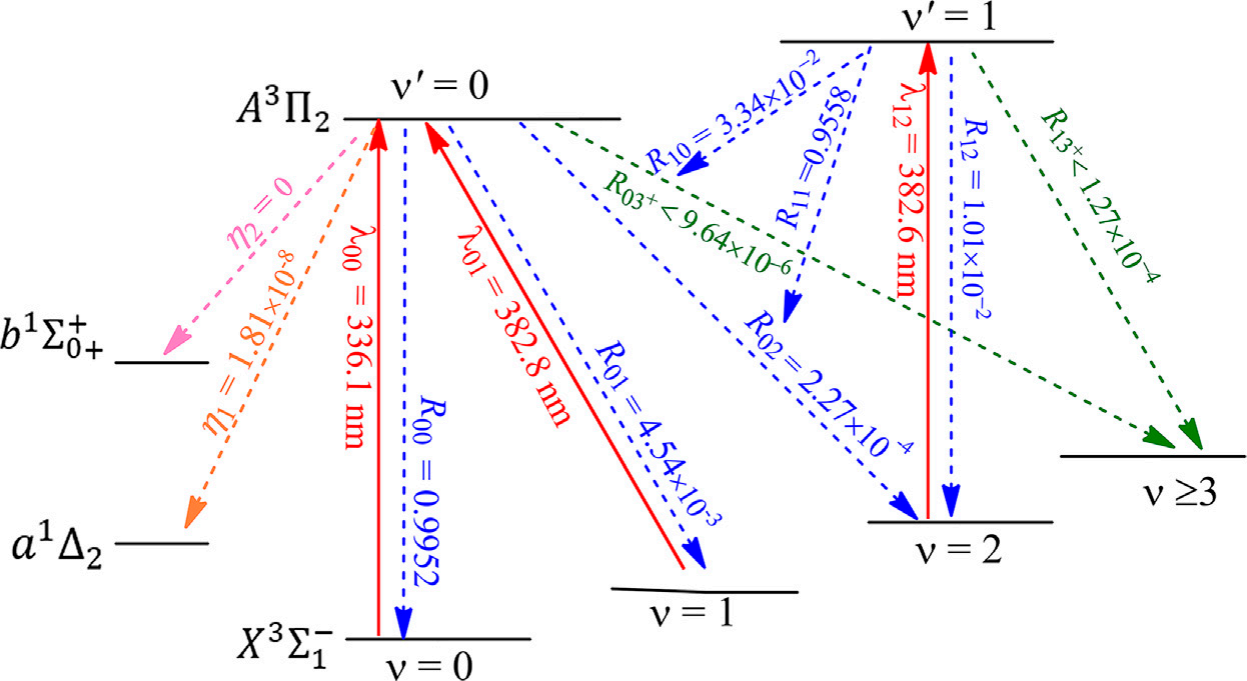
Constructed three-laser cooling scheme for NH using the $X^{3}\Sigma_{1}^{-}$ (*ν*) → $A^{3}\Pi_{2}$ (*ν*′) transitions. Solid arrows indicate laser-driven transitions at certain wavelengths $\lambda_{\nu^{\prime }\nu }$ . Dashed arrows indicate spontaneous decays from the $A^{3}\Pi_{2}$ (*v*′ = 0, 1) states with the calculated vibrational branching ratios. The rotational branching can be eliminated by driving the *J* = 1 → *J'* = 0 type transition (*J* is the rotational quantum number) for each vibrational level.

**FIGURE 9:**
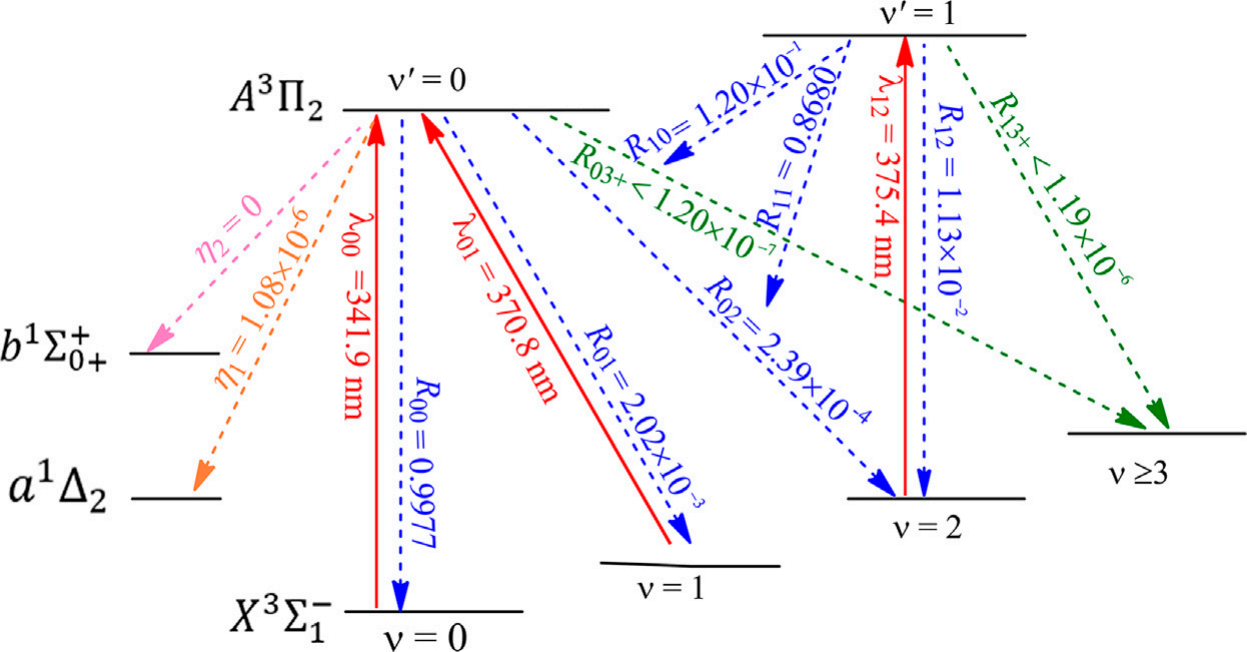
Constructed three-laser cooling scheme for PH using the $X^{3}\Sigma_{1}^{-}$ (*ν*) → $A^{3}\Pi_{2}$ (*ν*′) transitions. Solid arrows indicate laser-driven transitions at certain wavelengths $\lambda_{\nu^{\prime }\nu }$ . Dashed arrows indicate spontaneous decays from the $A^{3}\Pi_{2}$ (*v*′ = 0, 1) states with the calculated vibrational branching ratios. The rotational branching can be eliminated by driving the *J* = 1 → *J'* = 0 type transition (*J* is the rotational quantum number) for each vibrational level.

The constructed laser-cooling schemes for the production of ultracold NH and PH are presented in Figures 8 and 9, respectively. As seen in Figure 8, the laser for the main cycling may drive the $X^{3}\Sigma_{1}^{-}$ (*ν* = 0, J = 1) → $A^{3}\Pi_{2}$ (*ν*′ = 0, J′ = 0) transition of NH at the wavelength $\lambda_{00}$ of 336.1 nm (here J represents the rotational quantum number). According to the angular momentum and parity selection rules, the $A^{3}\Pi_{2}$ (J′ = 0) state can only decays to the initial $X^{3}\Sigma_{1}^{-}$ (J = 1) state, leading to the elimination of the rotational branching. In addition, another two lasers of 382.8 and 382.6 nm are used to recover the molecules falling to the $X^{3}\Sigma_{1}^{-}$ (*ν* = 1, 2) states of NH, further reducing the vibrational branching loss. So quasi-closed optical cycling can be achieved by using the scheme shown in Figure 8. Similarly, in Figure 9, the constructed scheme for PH take the $X^{3}\Sigma_{1}^{-}$ (*ν* = 0, J = 1) → $A^{3}\Pi_{2}$ (*ν*′ = 0, J′ = 0) transition as the main pump, the $X^{3}\Sigma_{1}^{-}$ (*v* = 1) → $A^{3}\Pi_{2}$ (*v*′ = 0) and $X^{3}\Sigma_{1}^{-}$ (*v* = 2) → $A^{3}\Pi_{2}$ (*ν*′ = 1) transitions as the first and second vibrational repump, respectively. The computed pump and repump wavelengths $\lambda_{00}$ , $\lambda_{01}$ and $\lambda_{12}$ are 341.9, 370.8 and 375.4 nm, respectively, which are all in the range of ultraviolet A (320 **∼** 400 nm) and can be produced with the frequency doubled Ti: sapphire semiconductor laser (Xing et al., 2018). The large $R_{00}$ values of NH (0.9952) and PH (0.9977) suggest that the $A^{3}\Pi_{2}$ (*ν*′ = 0) → $X^{3}\Sigma_{1}^{-}$ (*ν* = 0) transition of NH and PH has the largest possibilities, and the vibrational branching loss can be addressed through a reasonable laser cooling cycle process. The off-diagonal $R_{\nu^{\prime }\nu }$ of NH and PH have also been computed, and we use $R_{03^{+}}$ (here $3^{+}$ means *ν* ≥ 3) to evaluate the possibilities of unwanted decay channels for NH and PH. The negligible values of 9.64 × 10^–6^ (NH) and 1.20 × 10^–7^ (PH) mean that NH and PH can scatter at least 1.04 × 10^5^ (NH) and 8.32 × 10^6^ (PH) photons on average using the present schemes, respectively, which are enough to decelerate NH and PH in a cryogenic beam, in principle (Shuman et al., 2010).

After initial cooling and trapping stages, evaporative cooling is often used to bring molecules to quantum degeneracy or Bose-Einstein condensation. The possibility of evaporative cooling of NH has been investigated (Janssen et al., 2011; Janssen et al., 2013), however, recent accurate quantum calculations (Janssen et al., 2013) indicate that chemical reactions can cause more trap loss than inelastic NH + NH collisions, and evaporative cooling is not favorable for NH. As mentioned above, the laser cooling scheme constructed here allows for 1.04 × 10^5^ photons scattered for NH, which are sufficient for cooling to *µ*K temperatures. In addition, PH seems to be a better candidate than NH for laser cooling. So the present work indicates that the direct laser cooling method can be used to produce magnetically trapped ultracold NH/PH molecules, and it is expected that the subsequent evaporative cooling can be avoided.

## Conclusion

In this work, we identify two excellent ultracold molecular candidates from group VA hydrides using highly accurate *ab initio* method; in particular, NH and PH are identified as very promising laser cooling candidates, which satisfy all known criteria including the fourth one proposed in our recent work. Six low-lying Λ-S states of NH and PH are investigated with the SOC effects included. The agreement between our calculated spectroscopic constants and the available experimental data is excellent. We find that the locations of crossing point between the $A^{3}\Pi$ and Σ−5 states of NH and PH are higher than the corresponding *v*′ = 2 vibrational levels of the $A^{3}\Pi$ state indicating that the crossings with higher electronic states would not affect laser cooling. Meanwhile, the extremely small vibrational branching loss ratios of the $A^{3}\Pi_{2}$ → $a^{1}\Delta_{2}$ transition for NH and PH (NH: 1.81 × 10^–8^; PH: 1.08 × 10^–6^) indicate that the $a^{1}\Delta_{2}$ intermediate electronic state will not interfere with the laser cooling. Besides, the $b^{1}\Sigma_{0+}^{+}$ intermediate electronic state does not interfere since the $A^{3}\Pi_{2}$ → $b^{1}\Sigma_{0+}^{+}$ transition is forbidden. Consequently, we construct practical and efficient laser-cooling schemes for NH and PH on the basis of the $A^{3}\Pi_{2}$ → $X^{3}\Sigma_{1}^{-}$ transition. The calculated excitation energies to the $A^{3}\Pi$ state of NH and PH are 29,824.42 and 29,528.42 cm^−1^, respectively, which are in excellent accordance with the corresponding experimental data (NH: 29,807.4 cm^−1^; PH: 29,498.0 cm^−1^) (Huber and Herzberg, 1979). This enables us accurately predict the pump and repump wavelengths in laser cooling cycles. The Doppler temperatures for the main transition of NH and PH are 8.06 and 7.27 *µK*, respectively, whereas the recoil temperatures are 1.13 and 5.12 *µK*, respectively. The vibrational branching ratios $R_{\nu^{\prime }\nu }$ for the $A^{3}\Pi_{2}$ (*v*′ = 0) → $X^{3}\Sigma_{1}^{-}$ transition of NH and PH are shown to be highly diagonally distributed with $R_{00}$ being 0.9952 and 0.9977, respectively. The radiative lifetimes for the $A^{3}\Pi_{2}$ (*v*′ = 0) → $X^{3}\Sigma_{1}^{-}$ (*v* = 0) transition of NH and PH are extremely short (NH: 474 ns; PH: 526 ns). The constructed schemes allow for 1.04 × 10^5^ and 8.32 × 10^6^ photons scattered for NH and PH, respectively, which are sufficient for cooling to ultracold temperatures. Generally speaking, PH is a better candidate than NH for laser cooling. It is our hope that the present theoretical study will stimulate experimental interests in laser cooling NH and PH to the ultracold regime.

## Data Availability

The original contributions presented in the study are included in the article/Supplementary Material, further inquiries can be directed to the corresponding author.
