# Supplementary material for: Excellent Ultracold Molecular Candidates From Group VA Hydrides: Whether Do Nearby Electronic States Interfere?
Source: Front Chem. 2021 Dec 16;9:778292. doi: 10.3389/fchem.2021.778292 (PMC8716497; doi:10.3389/fchem.2021.778292)
Supplement: Supplementary file 1 [file Presentation1.pdf]

Supplementary Material

1 Supplementary Figures

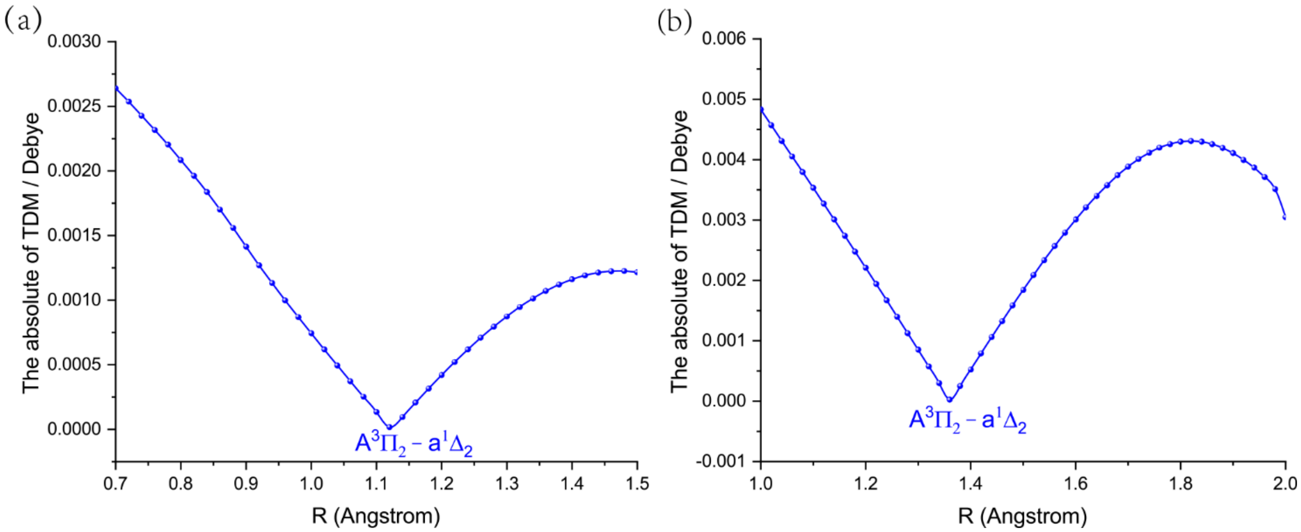

Figure S1. The transition dipole moments (TDMs) as a function of the interatomic distance (R) for the $A^3\Pi_2 - a^1\Delta_2$ transition of NH (a) and PH (b) at the icMRCI+Q level.

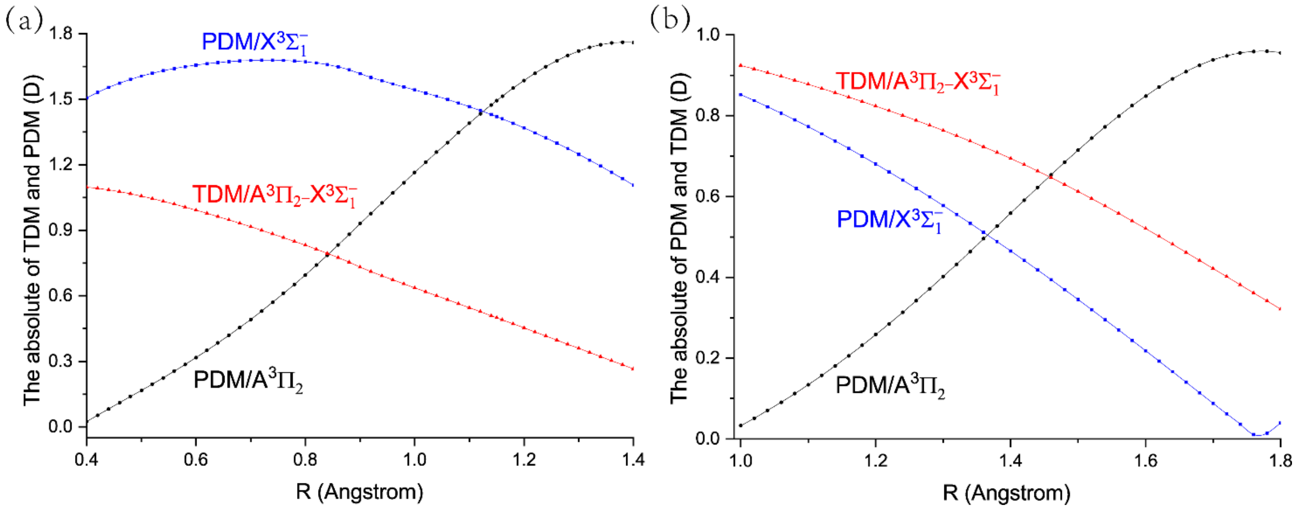

Figure S2. The permanent dipole moments (PDMs) and transition dipole moments (TDMs) as a function of the interatomic distance (R) for the $X^3\Sigma_1^-$ and $A^3\Pi_2$ states of NH (a) and PH (b) at the icMRCI+Q level.
